# Cutting-Edge Electrocatalysts for CO_2_RR

**DOI:** 10.3390/molecules28083504

**Published:** 2023-04-16

**Authors:** Nivetha Jeyachandran, Wangchao Yuan, Cristina Giordano

**Affiliations:** Department of Chemistry, Queen Mary University of London, Mile End Road, London E1 4NS, UK

**Keywords:** CO_2_RR, carbon dioxide capture, nanocatalysts, Cu-based electrocatalysis, metal electrocatalysts

## Abstract

A world-wide growing concern relates to the rising levels of CO_2_ in the atmosphere that leads to devastating consequences for our environment. In addition to reducing emissions, one alternative strategy is the conversion of CO_2_ (via the CO_2_ Reduction Reaction, or CO_2_RR) into added-value chemicals, such as CO, HCOOH, C_2_H_5_OH, CH_4_, and more. Although this strategy is currently not economically feasible due to the high stability of the CO_2_ molecule, significant progress has been made to optimize this electrochemical conversion, especially in terms of finding a performing catalyst. In fact, many noble and non-noble metal-based systems have been investigated but achieving CO_2_ conversion with high faradaic efficiency (FE), high selectivity towards specific products (e.g., hydrocarbons), and maintaining long-term stability is still challenging. The situation is also aggravated by a concomitant hydrogen production reaction (HER), together with the cost and/or scarcity of some catalysts. This review aims to present, among the most recent studies, some of the best-performing catalysts for CO_2_RR. By discussing the reasons behind their performances, and relating them to their composition and structural features, some key qualities for an “optimal catalyst” can be defined, which, in turn, will help render the conversion of CO_2_ a practical, as well as economically feasible process.

## 1. Introduction

**Figure 1:**
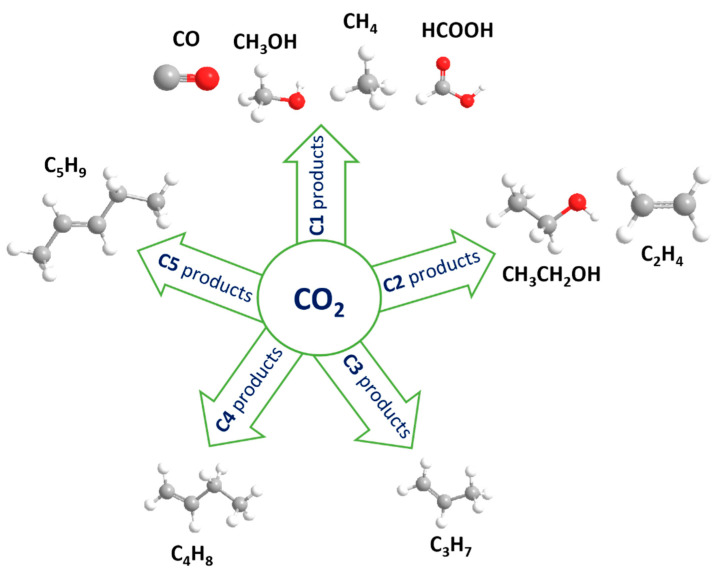
Overview of different products derived from the electrocatalytic CO_2_ reduction reaction (CO_2_RR).

The day-to-day increase in levels of CO_2_, predominantly produced by anthropogenic processes, poses serious environmental issues, and simply relying on nature (i.e., photosynthesis via plants) is no longer sufficient to stabilize the CO_2_ present in the air. The current CO_2_ concentration in the Earth’s atmosphere is approximately 417 ppm, and it has almost doubled since the 1760s [1]. This has led to devastating effects in terms of climate change, which in turn is causing melting glaciers, rising sea levels, destruction of natural habitats, and flooding, among others [2]. In order to improve the sustainability of our environment, besides limiting anthropogenic CO_2_ emissions, one effective solution to reduce CO_2_ levels relies on its capture and subsequent electrocatalytic conversion (also known as CO_2_ reduction reaction, or CO_2_RR) into value-added chemicals. These products can be very diverse, as schematically shown in Figure 1, and can be used as fuels, as versatile chemical intermediates, preservatives, pesticides, in cosmetics, and more [3].

Current research is focusing more and more on optimizing CO_2_RR to make it practical but also economically feasible. However, there are several challenges to face, the main one related to the energy costs of the reduction step (the CO_2_ molecule is thermodynamically stable, Δ_f_G_298K_ = −394 KJ/mol) [4,5], while achieving a conversion with high selectivity and efficiency.

To give an idea of these challenges, Table 1 reports the standard potential required to convert CO_2_ to various products.

Another challenge to face during the CO_2_ conversion process is the concurrent production of hydrogen, which commonly derives from the competing hydrogen reaction (HER). Clearly, this reaction reduces efficiency and needs to be suppressed to maximize the final yield and selectivity of the desired products.

These challenges can be mitigated by employing a suitable catalyst that is able to facilitate the breakage of the CO_2_ bonds, address the conversion toward specific products (selectively), suppress the HER, and remain stable throughout the process.

This review aims to present some recent studies on selected, most promising catalysts for CO_2_RR. By discussing the reasons behind their performance, we wish to delineate the next generation of electrocatalysts for rendering the conversion of CO_2_ an economically feasible process.

### Mechanism for Electrochemical Reduction of CO_2_

**Figure 2:**
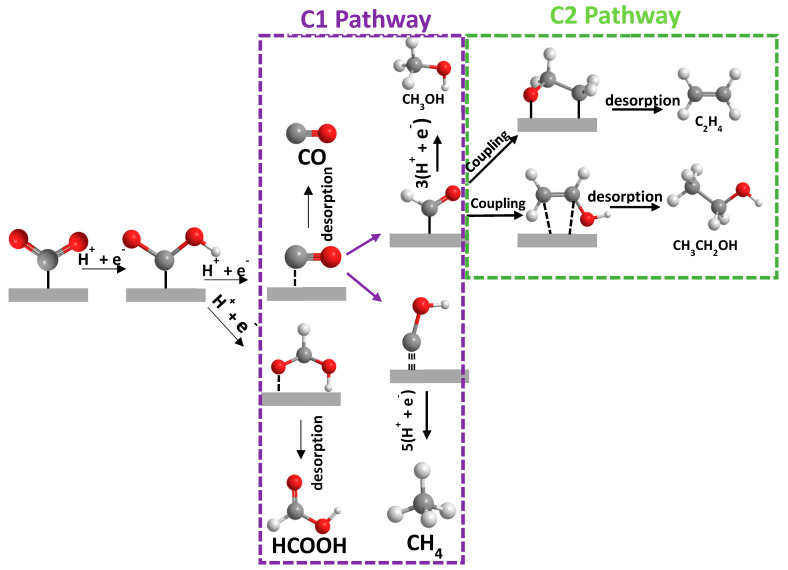
Scheme showing some possible pathways for the CO_2_ reduction to C1 and C2 products.

In general, the electrochemical reduction of CO_2_ in an aqueous solution is a multi-electron transfer process that enables CO_2_ to convert into several gaseous and liquid products, as shown in Figure 1 [7,8]. The final product obtained is dependent on several factors, such as the nature of the electrocatalyst (discussed in later chapters), and the electrolytic reaction conditions, including the electrolyte used [9], the applied potential, and the type of cell used for the setup (i.e., flow cell [10], H-cell [11]). To simply explain the mechanism in three steps: (1) CO_2_ adsorbs and interacts with surface atoms of the catalyst; (2) CO_2_ is activated, and the reduction proceeds with aid of the catalyst-initiated proton transfer to generate intermediates such as *CO_2_, *COOH, *CO, and others; and (3) the final product is desorbed, and the recovery of the catalyst surface takes place. These intermediates are crucial for CO_2_RR to lead to the final desired product (Figure 2). For instance, the generation of so-called C1 products involves the *CO_2_ intermediate interacting with a proton to form *COOH favoring the production of CO. Other intermediates, such as *CO, are invaluable to both the C1 and C2 pathways. To obtain C1 products, *CO can obtain a proton to generate *CHO intermediate, followed by three proton/electron transfers to form CH_3_OH. Moreover, the *CO intermediate can also participate in an additional step known as the C-C coupling step, leading to the production of C2 products [12].

## 2. Noble Metal-Based Nanosized Catalysts for CO_2_RR

A suitable catalyst for CO_2_RR must guarantee high selectivity while maximizing efficiency (yield/conversion). In this respect, noble metal-based catalysts such as Au [13], Ag [14], Pd [15], Rh [16], and Ir [17] were proved to have both excellent activity and high selectivity towards the formation of CO and formate (see Table 2).

**Figure 3:**
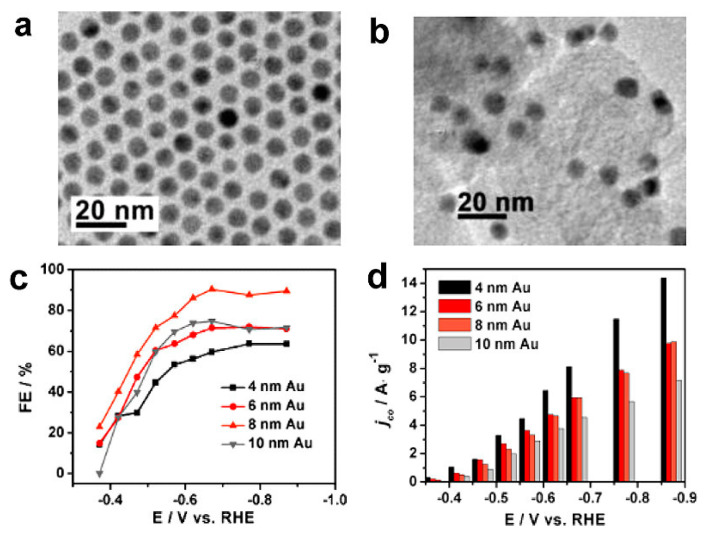
TEM images of (**a**) the 8 nm Au NPs and (**b**) the C-Au NPs (embedded in carbon matrix). (**c**) Potential-dependent FEs of the C-Au on electrocatalytic reduction of CO_2_ to CO. (**d**) Current densities for CO formation (mass activities) on the C-Au at various potentials. Reprinted (adapted) with permission from [13]. Copyright 2013 American Chemical Society.

For instance, Au nanoparticles (NPs) with sizes ranging between 4 and 10 nm [13] led to a selective electrocatalytic reduction of CO_2_ to CO, with FE 90% at −0.67 V (Figure 3). Furthermore, Au nanoparticles performed better than bulk Au; in fact, as observed by Kauffman et al. [19], bulk Au is barely active, showing a faradaic efficiency of 3% towards CO at a similar potential of −0.675 V vs. RHE. Interestingly, when Au NPs were embedded in a matrix of butyl-3-methylimidazolium hexafluorophosphate (BMIM-PF_6_), the FE towards CO increased by 7%, whilst simultaneously the H_2_ production was inhibited [13]. This suggests that the electrocatalytic performance for CO_2_ reduction is size-dependent but can be further enhanced in the presence of a functional support. Zhu et al. [13] attributed the better activity of the 8 nm nanoparticles to a higher number of edge sites (active sites for CO formation) than corner sites (active sites for hydrogen evolution). Lu et al. [14] studied the activity of nanoporous silver, comparing its performance with that of polycrystalline silver at different potentials. Similar to what observed by Zhu et al. [13], the nanoporous silver electrocatalyst was also more active, with a higher production of CO (FE of 92% at −0.6 V) compared to polycrystalline Ag (FE of 1.1% at the same potential). The study at lower overpotentials showed a decreasing efficiency toward CO formation and a greater production of hydrogen. On the other hand, higher overpotentials led to the formation of formate alongside CO, although hydrogen was still the primary product.

**Figure 4:**
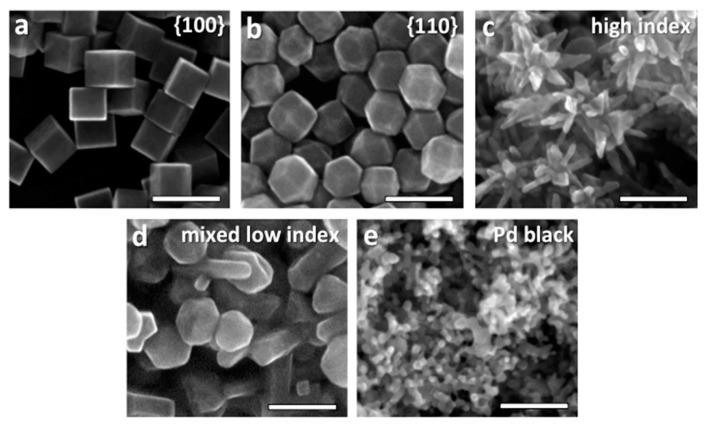
SEM images of Pd NPs used for electrode fabrication: NCs (**a**), RDs (**b**), BNPs (**c**), NPs with mixed low-index facets (**d**), and Pd black (**e**). Scale bar 100 nm. Reprinted (adapted) with permission from [15]. Copyright 2016 American Chemical Society.

Through computational studies, the role of surface morphology to maximize electrocatalytic performances was studied using palladium nanoparticles with sharp geometric features. Here, the predictions from DFT calculations were determined, and it was found that structures with more edges and grain boundaries have enhanced catalytic activity [15]. In this study, the Pd(211) plane exhibited the lowest energy barrier and, as a result, led to high catalytic activity towards formate production while suppressing CO formation. Following these theoretical predictions, Klinkova et al. [15] undertook experiments to develop Pd NPs with different shapes (see SEM images in Figure 4), and different types of stabilized facets: (100) plane-enclosed nanocubes (NC), (110) plane-enclosed rhombic dodecahedra (RDs), NPs with mixed low-index facets, and branched NPs enclosed by high-index facets (BNP). Klinkova et al. [15] showed experimentally that branched (BNP) Pd NPs surrounded by high index facets performed in agreement with theoretical predictions, reaching an impressive selectivity and a FE of 97% towards formate production at −0.2 V. Importantly, no CO production was detected.

These studies confirm that noble metal-based catalysts have high faradaic efficiency and selectivity, at moderate potentials toward CO and formate, and the main factors that influence the catalytic activity are the size of NPs, their potential-dependent selectivity, shape, and surface morphology. However, their main disadvantages cannot be ignored, i.e., their costs and scarcity, which make them less suitable for large-scale applications, along with their tendency to be poisoned.

## 3. Non-Noble Metal-Based Electrocatalysts for CO_2_RR

The costs related to CO_2_RR can be clearly lowered by using non-noble metal-based electrocatalysts. In this respect, several catalysts have been tested, including pure Sn [20], Ni [21], Fe [22], Zn [23], and Cu [24] (see Table 3). Loading non-noble metal nanoparticles on suitable carbon supports (e.g., carbon black or graphene) ensures a higher surface area and porosity, which eases CO_2_ transportation and subsequent reduction. Zhang et al. [20] discovered that 5 nm SnO_2_ NPs loaded onto graphene, rather than carbon black, enhanced activity [20], as evidenced by the higher FE achieved toward formate when a graphene support was used (93.6%), compared to carbon black (86.2%), both at −1.8 V, possibly due to the higher conductivity of graphene.

**Figure 5:**
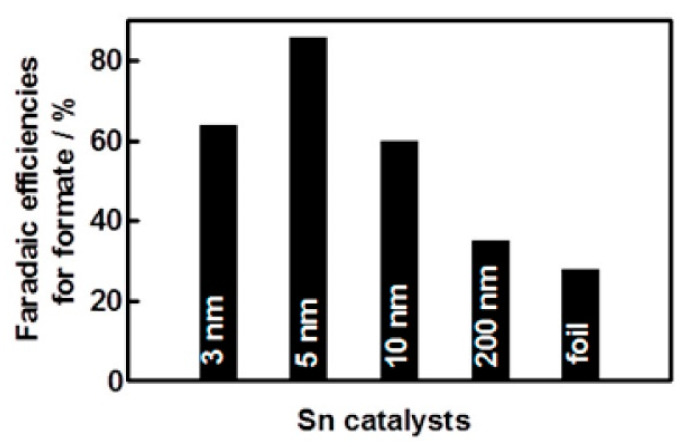
Particle size dependence of faradaic efficiencies for CO_2_ reduction to formate on Sn catalysts. Reprinted (adapted) with permission from [20]. Copyright 2014, American Chemical Society.

This study showed that the type of loading matrix used plays a key role by providing better conductivity, a stronger electronic interaction between the support and the metal nanoparticles and enhancing electronic donation. In this specific case, one can speculate that the stronger electron-donating ability of graphene, compared to carbon black, improves the CO_2_ reduction on the Sn surface. Unfortunately, hydrogen production was significant, thus lowering the overall efficiency. It is interesting to note, SnO_2_ NPs below 5 nm showed lower selectivity towards formate (to FE ~62%), even lower compared to Sn foil (FE of ~30%). It is evident that the particle size of the Sn catalysts has an influence on the CO_2_ reduction efficiencies (Figure 5). The maximum efficiencies achieved by the 5 nm nano-SnO_2_ catalyst were explained by the affinity between the surface-bound key intermediates and the catalyst, facilitating CO_2_ reduction. Remarkably, SnO_2_ NPs were found to be stable during electrolysis and had the ability to continuously produce formate for at least 18 h at 1.8 V. By comparison of the controlled potential electrolysis between the SnO_2_ NPs/carbon black and the SnO_2_ NPs/graphene, the steady state catalytic current density was twice as high for the latter, signifying the importance of support during CO_2_RR to improve performance. The stability of the catalyst after CO_2_RR was further confirmed by TEM images and LSV, indicating no significant changes regarding its morphology and catalytic property.

Nanoporous zinc oxide (ZnO) prepared via the hydrothermal method and thermal decomposition was also tested as a CO_2_RR alternative electrocatalyst by Jiang et al. [23]. Additionally, in this study, the main products were CO and H_2_. When the nanoporous ZnO was reduced to Zn, a greater faradaic efficiency of 92% toward CO formation at −1.66 V was achieved, also compared to commercial Zn (FE of 55.5%) at the same applied potential. These findings were adduced to the properties of nanoporous Zn, i.e., high surface area and high density of unsaturated, coordinated surface atoms. Hence, the high selectivity toward CO formation was explained by an increase in the activation of physiosorbed CO_2_ (a linear molecule) to chemisorbed CO_2_ (a bent molecule), caused by the surface defects and alkali metal promoted surfaces, facilitating the increased formation and stabilization of bent CO_2_^δ−^ intermediates on the coordination unsaturated surface atoms [23].

Based on the studies reported here, besides the mentioned advantages, non-noble metal-based catalysts showed a higher FE and selectivity towards CO and HCOO^−^. However, their performances were limited by their low selectivity and efficiency towards hydrocarbons and alcohol formation (so-called 2-C, 3-C, and 4-C products).

**Table 3:** Some examples of different non-noble metal-based electrocatalysts for CO_2_RR.

Electrocatalysts	Support	Main Product	FE (%)	Potential (V vs. RHE)	Reference
Carbon coated Ni NPs	N-doped carbon	CO	94	−0.7	[25]
Ni NPs encapsulated in N-doped carbon nanohybrid substrates	-	CO	93.1	−0.9	[26]
NiFe	2D MOF	CO	98.2	−0.5	[27]
Nanoporous NiSe_2_	-	CH_3_COOH	98.45	−0.25	[28]
ZnO nanowires	-	CO	91.6	−0.62	[29]
Co_hcp_ nanosheets	-	Ethanol	60	−0.4	[30]
Co NPs (5 nm)	Single layer nitrogen doped graphene	CH_3_OH	71.4	−0.90 V(SCE)	[31]
CoTe nanostructures	-	CH_3_COOH	87	−0.25	[32]
Fe-doped SnO_2_ electrode	-	HCOOH	41	−0.89	[33]
SnO_2_ NPs (<5 nm)	-	HCOO-	64	−1.21	[34]
CuO NPs/TiO_2_catalyst	TiO_2_	EtOH	~36	−0.85	[35]

## 4. Copper-Based Catalysts for CO_2_RR

Among non-precious metals that have been studied, copper-based materials are considered the best electrocatalyst choice for the conversion of CO_2_, also thanks to lower costs (compared to noble metals) and a relative abundance, with over 200 years of supply still left [36]. The abundance of Cu has a direct effect on its price, estimated at 0.20 GBP/oz (as of 29 March 2023) compared, for instance, to palladium or platinum, with prices estimated at around 1170 GBP/oz and 788 GBP/oz, respectively [37].

Carbon-based materials, including porous carbon, graphene, carbon nanotubes, and modified diamond, have shown different performances for CO_2_ reduction due to their different crystallinity, surface area, tunable chemical and physical properties, and good conductivity [38]. For instance, oxide carbon nanotubes can selectively convert CO_2_ into acetic acid with a faradaic efficiency of 71.3% [39]. However, from an economic perspective, the cost of these materials makes them not economically advantageous for practical applications; e.g., the price of 5 g of carbon nanotubes can range from GBP 59 to 259 [40]. Similarly, graphene and modified diamond are also expensive and thus less suitable for large-scale applications [41,42]. On the other hand, the Cu and Cu-based materials reported in this manuscript have been selected because they are readily available, inexpensive, and abundant. In addition to these advantages, the activity of Cu-based catalysts for electrocatalytic CO_2_ reduction has been established [43]. The unique 3d electronic structure of Cu allows a suitable amount of binding energy between the catalyst and the CO_2_ molecule for the activation of the CO_2_ to generate an activated *CO species. Furthermore, Cu-based catalysts are able not only to reduce CO_2_ simply into CO or short-chain hydrocarbons, but they also allow C-C coupling (by *CO dimerization), thus allowing the preparation of more complex C2+ products.

**Figure 6:**
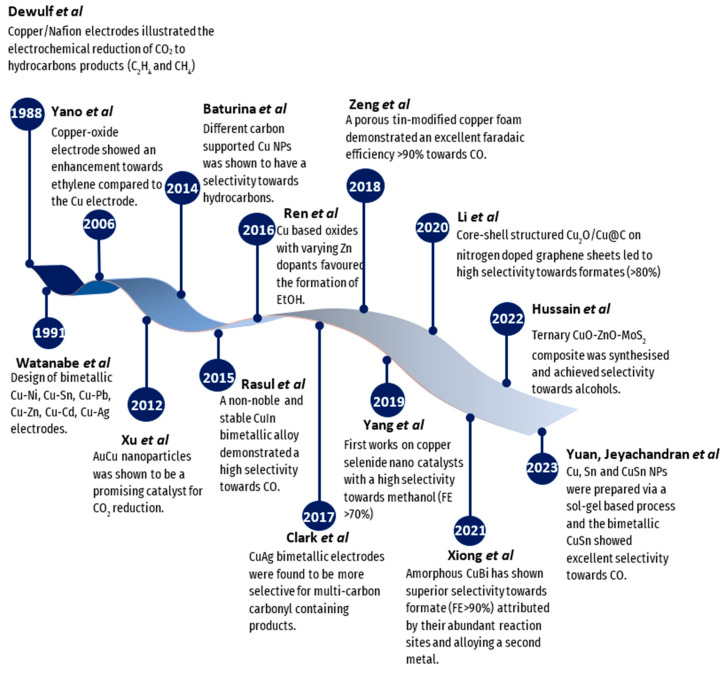
A timeline showing the development of Cu and Cu-based catalysts for the electrochemical reduction of CO_2_ into value-added fuels from 1988 to 2023 (from left to right, respectively) [44,45,46,47,48,49,50,51,52,53,54,55,56,57].

An overview of the timeline of the development of copper-based materials is reported in Figure 6.

**Figure 7:**
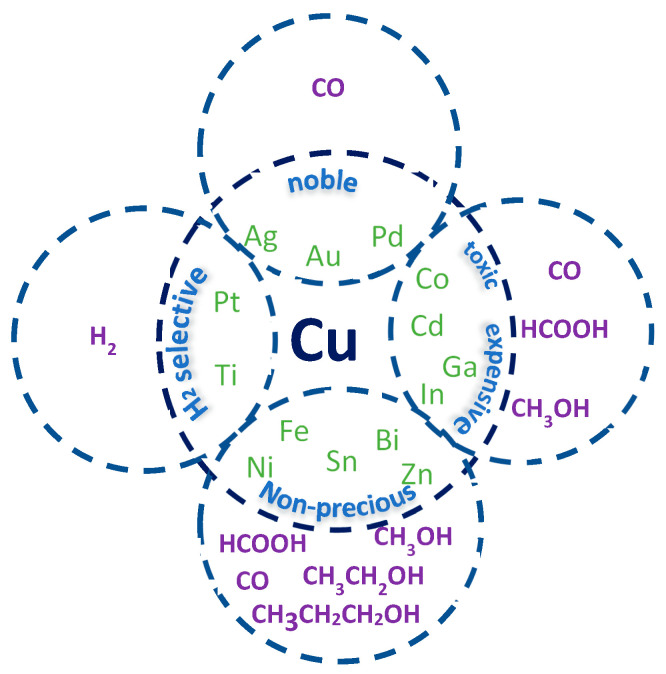
Scheme showing the different combinations of copper-based bimetallic catalysts so far reported in the literature for CO_2_ reduction, with their characteristics and general selectivity.

As for other metals discussed previously, pure copper also lacks selectivity towards specific products, which is why a second metal is usually incorporated to modify its structure, for instance, by metal doping, alloying, changing morphology (e.g., core/shell), adjusting crystallinity [58], and more, with the aim to exploit the resulting synergistic, strain, and alloying effects (Figure 7). The incorporation of one or more metals into the Cu structure has been shown to improve stability, increase selectivity and activity, and minimize energy consumption, overall leading to a more efficient CO_2_RR, as discussed in the following chapters.

There are several ways to synthesize Cu electrocatalysts; these include the use of microwave [59] and electron beam irradiation [60], laser ablation [61], thermal decomposition [62], in situ chemical synthetic routes [63], use of microemulsions [64], metal salt reduction [65], and sol-gel based processes [57], just to cite some. However, some of these methods are costly, lengthy, unable to control particles’ size, morphology, and/or crystallinity, or just not suitable for large-scale manufacture.

### 4.1. Copper-Based Alloys

Kim et al. [66] investigated the effect of the addition of gold to copper when preparing Cu-Au nanoparticles and found that the presence of gold influences the overall activity toward CO_2_RR. They showed that the more Au is incorporated into Cu nanoparticles, the more the formation of methane and ethylene declines, while the FE toward CO increases and H_2_ production is inhibited. By tuning the composition of Au-Cu bimetallic nanoparticles, the degree of stabilization of the intermediates on the nanoparticle surfaces is also affected, and, as a result, different products are favored. Kim et al. [66] also pointed out that the tested Au_3_Cu showed a high FE of 65% for CO, similar to pure Au NPs of similar sizes [13]. The outcome was elucidated considering the correlation between the nanoparticle’s composition and two other factors: (1) the electronic effect and (2) the geometric effect. The change in the electronic structure of the catalyst is influenced by the electronic effect on the binding strength of intermediates. The geometric effect is the local atomic arrangement at the active site. The way the active site is configured can affect the binding strength of intermediates. Therefore, both geometric and electronic effects must work synergistically to improve CO_2_ reduction [66]. Moreover, the stability of the Au_3_Cu catalyst was deduced from the total current as a function of time at −0.73 V vs. RHE. Despite stability in the current for 10 h, a steady decline in activity/selectivity towards CO production was observed following the first hour.

**Figure 8:**
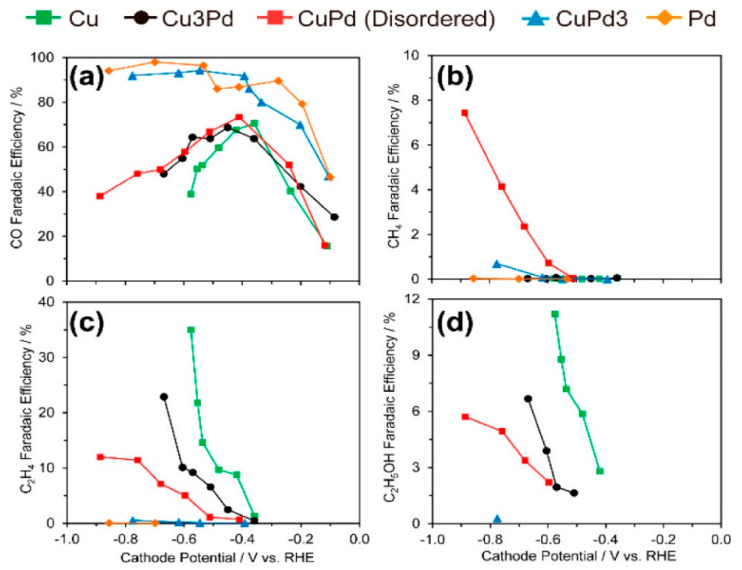
Faradaic efficiencies for (**a**) CO; (**b**) CH_4_; (**c**) C_2_H_4_; and (**d**) C_2_H_5_OH for catalysts with different Cu:Pd ratios: Cu, Cu_3_Pd, CuPd, CuPd_3_, and Pd. Reprinted (adapted) with permission from [67]. Copyright 2017 American Chemical Society.

Ma et al. [67] studied geometric effects and the way different mixing patterns can influence selectivity. The mixing patterns for the Cu-Pd catalyst were: ordered, disordered, and phase separated, with compositions ranging from 3:1 to 1:3. Here, it was observed that the more Cu present favored the formation of C2 products, confirming that composition has an influence on selectivity. Interestingly, the product formed depended on the type of mixing pattern used (Figure 8). The ordered CuPd presented the best selectivity for CO formation (FE of 80%), whereas the disordered CuPd showed poor selectivity for CO formation. However, the phase-separated CuPd demonstrated selectivity towards C_2_H_4_ and C_2_H_5_OH with FE values of ~50% and ~13%, respectively. Ma et al. [67] hypothesize that the electronic effects induced by the mixing of Pd with Cu resulted in different FE for different products. This was further investigated by collecting surface valence band photoemission spectra of all mixing patterns of Cu-Pd catalysts (including the corresponding monometallic ones), which showed that the phase-separated Cu-Pd catalyst has weaker binding between the CO intermediate and the catalyst surface, whereas monometallic Cu has a stronger CO binding. Despite these differences, both phases separated Cu-Pd and Cu NPs showed similar catalytic selectivity. This study showed that the geometric/structure effect had a more significant influence on selectivity than the electronic effect.

The conclusion was that when the Cu atoms were in close proximity to the Pd atoms, it favored alcohol and hydrocarbon formation, whereas when the Cu atoms were alternating with Pd atoms, it favored CO and CH_4_ formation. From this study, we can observe how adjustments over composition, alongside geometric and electronic effects, can enhance selectivity. Yet, alloying noble metals (i.e., Au, Pd) to copper is still relatively expensive and difficult to manufacture on a large scale.

### 4.2. Copper Alloyed with Non-Noble Metals

Alloying Cu with non-precious metals, such as Ni [68], Fe [69], Sn [70], Zn [71], Bi [55,72], and many others, can further lower the production price. Tan et al. [68] synthesized CuNi nanoparticles embedded in a three-dimensional (3D) nitrogen-carbon network and found outstanding performances with regards to CO_2_RR. It was observed that CuNi NPs can selectively convert CO_2_ into CO (FE 94.5%) at low potentials and outperform their corresponding mono-metal catalysts. The high selectivity was explained by the 3D nitrogen-carbon network improving the CO_2_ adsorption capacity of the system (i.e., more CO_2_ being adsorbed on the surface of the catalyst) and enhancing the selectivity towards specific products, as well as improving the stability of the catalyst. The performance of the CuNi catalyst was improved with regards to the catalyst’s stability, demonstrating a stability of constant potential electrolysis for over 38 h at −0.6 V vs. RHE. From a structural and compositional point of view, the catalyst remains unchanged after CO_2_RR, as confirmed by XRD and TEM. However, even though catalytic performances were outstanding, the synthesis of CuNi NPs embedded in the nitrogen-carbon network is very time-consuming, requiring a total time of about 50 h. 

#### 4.2.1. Copper-Based Core/Shell Systems

**Figure 9:**
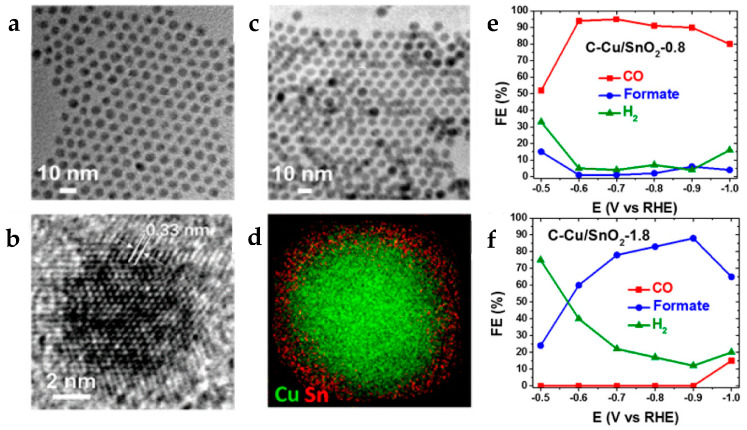
TEM images of (**a**) 7 nm Cu NPs and (**b**) Cu/SnO_2_ (0.8 nm shell thickness) NPs. (**c**) HR-TEM image of a representative Cu/SnO_2_ (0.8 nm shell thickness) NP. (**d**) EELS elemental mapping of Cu and Sn in a 0.8 nm Cu/SnO_2_ NP. Reduction potential-dependent FE’s for electrochemical reduction of CO_2_ measured on (**e**) C-Cu/SnO_2_-0.8 and (**f**) C-Cu/SnO_2_-1.8 catalysts. Reprinted (adapted) with permission from [70]. Copyright 2017 American Chemical Society.

Changing structure and morphology can also lead to improved performance. Thus, alongside copper alloys, core/shell systems were studied. Compared to alloys, core-shell structures enable additional control over the core size and the thickness of the shell, which can influence the electrochemical reduction of CO_2_. Li et al. [70] synthesized Cu core/SnO_2_ shell NPs using the seed-mediated method, where the synthesis of Cu NPs is followed by the decomposition of Sn(acac)_2_ [70]. One advantage of this method is that it allows precise control over size and leads to core/shell structures with adjustable shell thickness [73]. The core copper NPs were kept at a controlled size of 7 nm, and the thickness of the SnO_2_ shell was changed by adjusting the amount of Sn(acac)_2_ added, with ±1 nm final precision. This study showed that both activity and selectivity were thickness dependent (Figure 9). In fact, when the thickness of the SnO_2_ coating was above 1 nm, the favored product was formate (FE of 85% at −0.9 V). Whereas, when the SnO_2_ coating was below 1 nm, the main product was CO (FE 93% at −0.7 V). Unfortunately, the drawback of this method is the instability of the seeds [74]. Yet, Cu/SnO_2_ (0.8 nm shell) demonstrated good stability for 10 h at −0.6 V, and the core/shell structure remains intact even after CO_2_RR, as confirmed by CV data (before and after CO_2_ test showing similar surface redox potential) and TEM elemental mapping.

Similarly, Zhang et al. [75] explored Cu@Ag core/shell nanoparticles and also found that tuning the thickness of the shell was a crucial contributor to the selectivity of CO_2_RR. Optimized performances were observed for Cu@Ag_2_ NPs with FE of 67.6% and 32.2% toward C2 products and ethylene at −1.1 V vs. RHE, respectively. Increasing the thickness of the Ag shell to Cu@Ag_3_ or Cu@Ag_4_ resulted in an increase in CO formation (with similar selectivity as pure Ag NPs [14,76]), while C2 products decreased. Again, selectivity was shown to be thickness-dependent but also influenced by the choice of metal. In fact, while the previous study on Cu@Sn showed that CO and formate were mostly preferred, the Cu@Ag system favours the production of C_2_H_4_ and other C2 compounds (including acetate and ethanol). One explanation for the observed difference in selectivity can be ascribed to the core/shell structure of the Cu@Ag, which can function as “a tandem catalyst”, i.e., CO_2_ is first attached to the Ag shell, activated, then reduced into CO, followed by its conversion into C2+ products on the Cu core. The stability of the Cu@Ag electrocatalyst was tested by performing chronoamperometry measurements (i.e., constant applied potential as a function of time under CO_2_ reaction conditions). The measurements showed excellent durability with a steady current density and, more importantly, consistent production of C_2_H_4_ (FE maintained about ~30%) for 14 h at an applied potential −1.1 V vs. RHE for the Cu@Ag_2_ catalyst. To prove the stability of the catalyst here, TEM and XRD were performed after CO_2_RR, showing no changes in the composition or morphology of the catalyst. Although desirable products were formed when alloying Ag with Cu, Ag is an expensive, precious metal, making it not practical for large-scale applications.

**Figure 10:**
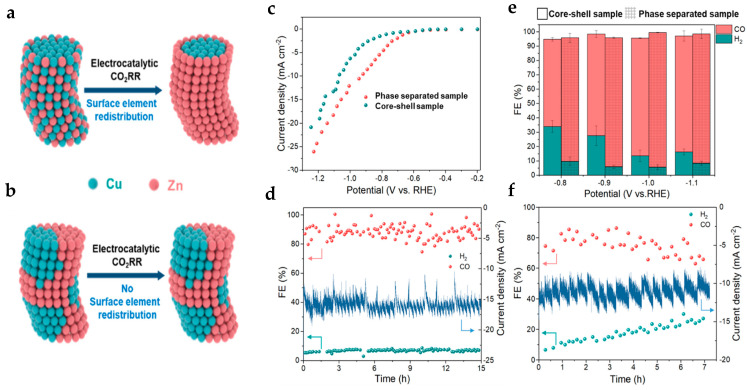
Schematic illustration of surface element redistribution in (**a**) core–shell and (**b**) phase-separated samples. Electrochemical CO_2_ reduction performance. (**c**) Linear sweep voltammetry curves of phase-separated and core–shell samples in a CO_2_-saturated 0.1 M KHCO_3_ solution. (**e**) FE of the main products of core–shell and phase-separated samples. The long-term stability test of (**d**) phase-separated and (**f**) core–shell samples. Reprinted (adapted) with permission from [77]. Copyright 2022, American Chemical Society.

A different approach explored the influence of the elemental spatial distribution in bimetallic CuO_x_-ZnO nanowires after an in situ electrochemical reduction by Wan et al. [77]. Interestingly, they discovered that the phase-separated structural distribution possesses better activity, with higher faradaic efficiency towards CO (>90%), and greater stability over time compared to core/shell structures (FE > 80%) (Figure 10). Unfortunately, the FE declines over time as competitive H_2_ production increases [77]. Also interestingly, regarding stability, the phase-separated catalyst demonstrated longer durability with a constant rate of FE_CO_ and a current density stabilized at 16 mA^−2^ for 15 h. In comparison, the CuZn core-shell structure was relatively unstable for long durations, and after about 2.5 h, a decline in FE_CO_ and a rise in H_2_ production was observed (see Figure 10e,f). In fact, the CuZn phase-separated was more stable than CuZn core/shell, this was suggested by the observation that element redistribution occurred (after 20 min of the CO_2_RR) possibly due to the strain on the Zn atoms, with consequent precipitation of Zn, resulting in morphological changes, such as the appearance of dendritic structures (shown by SEM images) for the core/shell sample. The drastic change in morphology for the core/shell after CO_2_ testing was reasoned to account for the observed inactivation in CO_2_ performances. On the other hand, CuZn phase-separated reported no morphological transformation or elemental redistribution to be seen after CO_2_RR. Therefore, we can clearly conclude here that the type of structure has an influence on the final stability and catalytic performance [77].

Other studies (e.g., Ren et al. [50]) on Cu-Zn systems for CO_2_RR have found that introducing Zn dopant as a co-catalyst can assist copper catalysts to selectively convert CO_2_ into ethanol. In fact, previous studies have shown that carbon monoxide (CO) can be reduced into ethanol on Cu NPs [78]. It was then showed that the presence of Zn dopants can increase the CO-producing sites in situ on oxide-derived copper catalysts during CO_2_RR for selective ethanol formation. It seems that the more Zn is added, the maximum faradaic efficiency towards ethanol increases (FE 29.1%), whereas an excess of Zn leads to a decrease in ethanol formation. Further studies were performed on other co-catalysts such as Ag and Ni on copper-based catalysts (where Ag is selective for CO formation and Ni is inactive towards CO_2_RR), but they showed a much lower selectivity compared to Zn towards ethanol(Zn > Ag > Ni). A suggestion for Ag having a low selectivity towards ethanol compared to Zn was deduced from their different CO binding strengths [79]. Whilst EtOH is a desirable product obtained from CO_2_, the catalytic stability of the Cu_4_Zn catalyst for EtOH production was a minimum of 5 h. In addition, significant morphological changes (confirmed by SEM) were observed following the first hour of CO_2_ reduction, which were attributed to the relief of structural strain during the reduction process [50]. SAED patterns from TEM investigation confirmed that CuZn was no longer an alloy, but rather a phase segregation of Cu and Zn was seen after the reduction process.

Other core/shell catalysts prepared and tested for CO_2_RR are reported in Table 4 with their synthetic methods. Generally, it is observed that they tend to favor C1 products, i.e., CO and formate. This selectivity can be however changed by tuning the thickness of the shell, which seems to influence the final product more than the choice of the metal.

#### 4.2.2. The Influence of Supports

The electrochemical reduction of CO_2_ necessitates that the Cu-based catalyst be placed on a conductive material to maximize electron transport. Common supports are carbon black [13], carbon nanotubes [87], and graphene [88]. To investigate the role of support, Li et al. [88] loaded Cu NPs onto three different matrices, namely Kejen black EC-300 carbon, graphene oxide, and pyridinic-N-rich graphene (p-NG). When the copper NPs deposited onto Kejen black EC-300 carbon were tested, the conversion of CO_2_ to C_2_H_4_ was below an FE of 10%. However, when the Cu NPs were placed onto the p-NG support, the selectivity and efficiency towards C_2_H_4_ increased. The high selectivity towards C_2_H_4_ was explained as a result of the synergistic effect between the p-NG support and Cu NPs. It was suggested that since the p-NG structure is a strong Lewis base, it can allow the protons to concentrate around the Cu and facilitates CO_2_ to interact with Cu, resulting in CO_2_ reduction and C-C coupling for the formation of ethylene. However, when p-NG alone (as a catalyst) was tested for CO_2_RR, it was found that p-NG favors the formation of formate and H_2_, thus highlighting the importance of the interaction between the Cu NPs and p-NG support.

**Figure 11:**
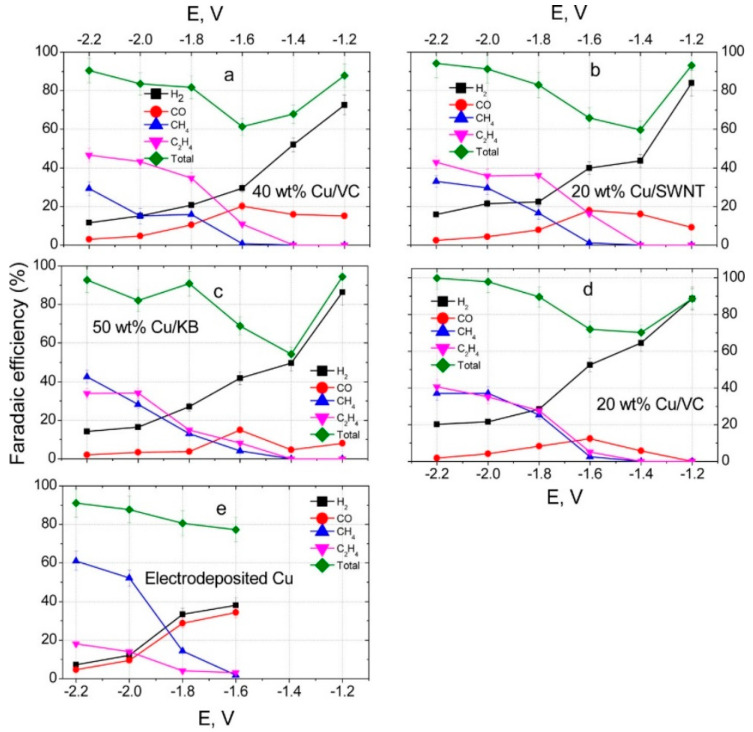
Faradaic efficiencies vs. potential for H_2_ (solid black squares), CO (solid red circles), CH_4_ (solid blue triangles), and C_2_H_4_ (magenta solid upside-down triangles) generation at thin films of (**a**) 40 wt % Cu/VC, (**b**) 20 wt % Cu/SWNT, (**c**) 50 wt % Cu/KB, (**d**) 20 wt % Cu/VC, and (**e**) electrodeposited Cu. Green diamonds: total faradaic efficiencies for H_2_, CO, CH_4_, and C_2_H_4_ generation. Reprinted (adapted) with permission from [48]. Copyright 2019, American Chemical Society.

Baturina et al. [48] also explored carbon-supported Cu nanoparticles for CO_2_RR, in particular: Vulcan Carbon (VC), Ketjen Black (KB), and Single-Wall Carbon Nanotubes (SWNTs), where Cu was either merely supported or electrodeposited. The carbon-supported Cu nanocatalysts achieved a selectivity toward higher ratios of C_2_H_4_:CH_4_ compared to the electrodeposited (and smoother) Cu films (Figure 11). The high selectivity towards C_2_H_4_ was ascribed to the presence of so-called ‘rough surfaces’, which present more corners, edges, and defects, along with smaller particle sizes, than the smooth surface of the electrodeposited Cu film. However, the reasoning behind the different stability patterns observed for different catalysts was unexplained. Also according to this study, the production of C_2_H_4_ and CH_4_ does not significantly change with time, but H_2_ production increases due to changes on the surface. The choice of support clearly has an influence on catalytic performances yet using supports like single-wall carbon nanotubes (SWNTs) can limit large-scale applications due to their costs (ranging from 125–300 USD/gram) [89].

#### 4.2.3. Crystalline Versus Amorphous Cu Nanoparticles

So far, research has mainly focused on crystalline Cu-based catalysts, however it was observed that amorphous Cu-based catalysts might perform better toward CO_2_ conversion compared to crystalline Cu nanoparticles, due to their lower-coordinated atoms, increased number of defects, and reactive sites (Table 5).

Duan et al. [58] synthesized amorphous Cu nanoparticles and tested them for electrochemical reduction of CO_2._ They found that the FE towards HCOOH, C_2_H_5_OH, and CO were 37%, 22%, and 5.8%, respectively, at −1.4 V, while crystalline Cu NPs achieved a lower FE towards HCOOH and C_2_H_5_OH at much lower potentials (compared to amorphous Cu nanoparticles). More notable, the amorphous Cu NPs exhibited a higher stability for up to 12 h at a constant applied potential of −1.4 V vs. RHE. In addition, the stability of amorphous Cu NPs was seen to be more attractive as negligible changes in composition, morphology, and catalytic property were identified by XRD, TEM, and LSV characterizations, respectively. In contrast, crystalline Cu NPs after 12 h of electrolysis showed poor stability t after 12 h due to an increase in crystallinity, agglomerated particles, as observed by XRD, TEM, and LSV, respectively. Duan et al. [58] suggested the superior performances showed by the amorphous Cu NPs relate to a higher electrochemical active area, arising from the abundant active sites, originating from the distinct electronic structure and intrinsic chemical heterogeneity on the surface of the amorphous Cu. The cyclic voltammetry (CV) data showed that the higher the electrochemical double layer capacitance (EDLC), the higher the electrochemical active area, which led to more active sites and allowed a greater adsorption of CO_2_ compared to crystalline Cu NPs. 

Table 6 reports an overview of some Cu-based catalysts tested in CO_2_RR.

## 5. Conclusions

In the present review, selected electrocatalysts for the conversion of CO_2_ (referred to as CO_2_RR) into added-value chemicals (including carbon monoxide, formic acid, methane, and ethanol) were discussed. Special emphasis was given to electrocatalysts with improved stability and high selectivity toward specific products over systems with high faradic efficiency (FE) only.

Amongst the various electrocatalysts discussed in this review, more focus was given to Cu and Cu-based catalysts, for these systems (more than others), can be tailored to produce a wider range of multi-carbon-based products, whilst simultaneously ensuring large-scale manufacture.

While, studies on noble metals (e.g., Au, Ag) confirmed good activity, high faradaic efficiency, and selectivity toward CO_2_RR, yet the main products were usually CO and formate. Even though it was shown that the applied potential can affect selectivity, the production of more complex hydrocarbons is still a challenge when using noble metals. In addition, these systems are in general costly, scarce, and have a tendency to undergo poisoning, making them unsuitable for large-scale applications.

From this perspective, non-noble metal-based catalysts are a preferable choice, thanks to their lower costs and readiness, but they can still show low selectivity and efficiency, especially towards hydrocarbons and alcohol formation (the so-called 2-C, 3-C, and 4-C products). Nonetheless, performances can be enhanced by using nanoparticles, rather than bulk systems. Nanoparticles’ size was shown to be one of the main factors influencing the system’s catalytic activity, alongside the particles’ shape, surface arrangements, degree of crystallinity, and general surface order/disorder of the final structure. Even surface roughness was shown to play a role, leading to different catalytic performances; i.e. CO_2_ conversion was shown to be favoured by the presence of defects, corners, and edges, commonly found in rough surface-based catalysts, which resulted in better catalytic performances compared to smooth surfaces.

The importance of moderate potentials (0.6–0.7 V) was also shown for non-noble metals, e.g., on Sn catalysts. Adjustments over composition and atomic arrangement, alongside geometric and electronic effects, can enhance selectivity, stability, and efficiency. Studies on CuZn catalysts, for instance, showed how some factors, such as metal element distribution and the use of co-catalysts can influence selectivity. It was also shown that tailoring morphologies may have a significant effect. Studies on core-shell structures (e.g., on CuSnO_2_) showed that selectivity can be changed by tuning the thickness of the particles’ shell, which in some cases influenced the final product more than the choice of the metal itself.

The important role of functional support was also discussed. Supports can serve to improve conductivity and general electron transport in the final system, can improve the diffusion path, as well as enhance stability. For instance, it was observed that using a more structured matrix, such as graphene, led to better overall performance than using mere amorphous carbon.

In our discussion, we have also pointed out how multi-metallic systems (e.g., bi-metallic or alloys) showed better electrocatalytic activity, outperforming the pure metals, thanks to synergistic effects, possibly resulting from geometric and electronic factors, and an increased number of active sites. These so-called “tandem catalysis” also showed better selectivity, along with higher activity.

Clearly, rather than a screening approach, a dedicated study is needed to develop outperforming catalysts. The studies undertaken so far have shown that the best choice relies on multi-metallic systems with a suitable choice of metals, composition, size, and atomic arrangement to maximize efficiency and selectivity towards multi-carbon-based products. Special attention should also be paid to the synthetic method and/or preparation technique, for it is surely a contributing factor to the final performance, whilst also considering the eco-friendliness of the process, total time, and final cost.

## Figures and Tables

**Table 1:** The standard potentials for the electrochemical reduction of CO_2_ [6].

Reaction	*E*^0^/V vs. SHE
CO_2_ + 2H^+^ + 2e^−^ → CO + H_2_O	−0.53
CO_2_ + 2H^+^ + 2e^−^ → HCOOH	−0.61
CO_2_ + 6H^+^ + 6e^−^ → CH_3_OH + H_2_O	−0.38
CO_2_ + 8H^+^ + 8e^−^ → CH_4_ + 2H_2_O	−0.24
2CO_2_ + 12H^+^ + 12e^−^ → C_2_H_4_ + 4H_2_O	−0.34

**Table 2:** Summary of noble metal-based electrocatalysts for CO_2_RR.

Electrocatalysts	Main Product	FE (%)	Potential (V vs. RHE)	Reference
Au nanoparticles (8 nm)	CO	90	−0.67	[13]
Nanoporous Ag	CO	92	−0.60	[14]
Branched Pd nanoparticles ~100 nm)	HCOOH	97	−0.20	[15]
Pd nanoparticles (3.7 nm)	CO	91.2	−0.89	[18]
Rh/Al_2_O_3_	CH_4_	-	-	[16]
(POCOP)Ir(H)(HSiR_3_)	CH_4_	-	-	[17]

**Table 4:** Comparison of core/shell-based systems for CO_2_RR.

Composition	Method	Advantage/Disadvantage of the Synthetic Method	Electrolyte	FE (%)	Potential (V vs. RHE)	Main Product	Ref
Cu/Pb nanocrystals	Chemical reduction	Advantages: large-scale production and no chemical purification is required. Disadvantages: size distribution	1 M KOH	~33	1.3	C2+ Liquid	[80]
Ag_3_Sn@SnO_X_ NPs	Seed growth and galvanic displacement method	Advantage of seed growth: good control of NP size [81]. Advantages of the galvanic displacement method: fine particle and morphology control [82].	0.5 M NaHCO_3_	~80	−0.8	Formate	[83]
Cu@SnO_2_ 0.8 nm	Seed-mediated method via the decomposition of tin acetylacetonate	Disadvantage: the instability of the seeds [74].	0.5 M KHCO_3_	93	−0.7	CO	[70]
Cu@SnO_2_ 1.8 nm				85	−0.9	Formate	
Cu@Ag_2_ core/shell NPs	Two step reduction process		1.0 M KOH	32.2	−1.1	C_2_H_4_	[75]
Cu/In_2_O_3_	Seed-mediated method via the decomposition of In(acac)_3_		0.5 M KHCO_3_	~70	−0.7	CO	[84]
Au-Fe core-shell NPs	Solvothermal method	Advantage: good control in both liquid-phase and multiphase chemical reactions. Disadvantages: expensive and requires high temperatures [85].	0.5 M KHCO_3_	97.6	−0.4	CO	[86]

Despite these promising results, the yield towards the preparation of more complex products remains a challenge, i.e., the so-called C2 (C_2_H_4_, C_2_H_5_OH), C3 (C_3_H_6_, C_3_H_7_OH), and C4 (C_4_H_7_, C_4_H_8_O) products.

**Table 5:** Comparison of amorphous bimetallic electrocatalysts for CO_2_RR in the literature.

Composition	Synthetic Method	Electrolyte	FE (%)	Potential (V vs. RHE)	Main Product	Ref
Sn1-xBix alloy NPs with natively bi-doped amorphous SnO_x_ nanoshells	Co-reduction	0.5 M KHCO_3_	95.8	−0.88	Formate	[90]
Amorphous CuBi	Electrodeposition method	0.5 M KHCO_3_	>94.7	−1.0	Formate	[55]
Crystalline Cu@amorphous SnO_2_	One pot wet chemical method	0.1 M KHCO_3_	70	−1.45	Formate	[91]

**Table 6:** The various Cu-based nanocatalysts used for CO_2_RR.

Composition	Support (If Any)	FE (%)	Potential (V vs. RHE)	Main Product	Advantages/Disadvantages	Ref
Cu NPs R5		11	−1.5 V vs. Ag/AgCl	CO	Green sol-gel methodology. No use of additives, supports, or co-catalysts.	[57]
Sn NPs R5		13	−1.5 V vs. Ag/AgCl	CO	Green sol-gel methodology.	[57]
CuSn alloy NPs R5		19	−1.5 V vs. Ag/AgCl	CO	Good control of composition and structure for the formation of nano-alloy.	[57]
Cu@Sn-B core/shell NPs R5		51	−1.5 V vs. Ag/AgCl	CO	Good control of composition and structure for the formation of core-shell.	[57]
Sn@Cu-C core/shell NPs R5		32	−1.5 V vs. Ag/AgCl	CO	Good control of composition and structure for the formation of core-shell.	[57]
Cu-Bi NPs	-	70.6	−1.2	CH_4_	High potential required.	[92]
Cu-Cd	-	84	−1.0	CO	Cd is toxic.	[93]
Cu-Co NPs	Carbon nanofibers	68%	−0.8	CO	Co is toxic.	[94]
CuCo_2_Se_4_		98	−0.25	CH_3_COOH	The presence of a Co center reduces catalyst poisoning.	[95]
CuCo NPs	-	>85	−1.1	H_2_	Co is toxic and favors HER	[96]
CuFe	Porous N-doped graphitic carbon	96	−0.3	CO	Fe is inexpensive and abundant.	[69]
CuIn	Carbon nanotubes	88.1	−0.6	CO	In is unstable, scarce, and expensive.	[97]
CuNi NPs	Nitrogen-carbon network	94.5	−0.6	CO	Ni is cheap and non-toxic.	[68]
CuNi nanosheet	-	92	−0.5	CO	CuNi nanosheet is air-stable and behaves similarly to Au and Ag.	[98]
CuSe_2_ NPs		84	−0.6	EtOH		[99]
CuSn NPs Sn NPs	Nitrogen Doped Graphene	60 54	−1.0	HCOO- and CO	Sn is abundant and inexpensive.	[100]
Porous CuTi	-			H_2_	Favors HER.	[101]
CuPt nanocrystals	-		−1.6	H_2_	Pt is expensive. Strong affinity between Pt and CO*. Hence, favoring HER over CH_4_ production.	[102]
CuO_x_-ZnO phase-separated nanowires CuOx-ZnO Core-shell	-	94 82	−1.0	CO	Zn is inexpensive.	[77]
Oxide-derived Cu_4_Zn	-	29.1	−1.05	C_2_H_5_OH		[50]

## Data Availability

Data are available from the authors.
